# Evaluation of an automatic HPLC analyser for thalassemia and haemoglobin variants screening

**DOI:** 10.1155/S1463924695000125

**Published:** 1995

**Authors:** R. Galanello, S. Barella, D. Gasperini, L. Perseu, E. Paglietti, C. Sollaino, L. Paderi, M. G. Pirroni, L. Maccioni, A. Mosca

**Affiliations:** 1 Istituto di Clinica e Biologia dell'Età Evolutiva Università degli Studi di Cagliari Via Jenner s/n Cagliari 09100 Italy; 2 Dipartimento Scienze e Tecnologie Biomediche Univcrsità degli Studi di Milano Via Olgettina, 60 Milan 20132 Italy; 3 Istituto di Clinica e Biologia dell'Età Evolutiva Ospedale Regionale Microcitemie USL 21 Università degli Studi di Cagliari Via Jenner s/n Cagliari 09100 Italy

## Abstract

In this paper the authors report the evolution of a new automatic HPLC analyser for screening haemoglobinopathies. HbA_2_ and F determinations are accurate and reproducible. The analysis time is short (6.5 min) and there is a good separation between the HbA_2_ values of β-thalassemia carriers from normals and α-thalassemia carriers, with no overlap between these groups. In addition, the system is also able to detect and quantitate most of the haemoglobin variants, particularly those (HbS, HbC, HbE and Hb Lepore) able to interact with β-thalassemia and could make haemoglobin electrophoresis unnecessary in all samples. The ease of operation and the limited technical work make this system especially suitable for laboratories with a high workload and allow the cost of screening to be reduced.


Journal of Automatic Chemistry, Vol. 17, No. 2 (March-April 1995), pp. 73-76

### Evaluation of an automatic HPLC analyser for thalassemia and haemoglobin variants screening

**R. Galanello, S. Barella, D. Gasperini, L. Perseu, E. Paglietti, C. Sollaino, L. Paderi, M. G. Pirroni, L. Maccioni and A. Mosca**

*Istituto di Clinica e Biologia dell'Età Evolutiva, Università degli Studi di Cagliari, Via Jenner s/n, 09100 Cagliari, Italy*

*In this paper the authors report the evolution of a new automatic HPLC analyser for screening haemoglobinopathies. $HbA_2$ and F determinations are accurate and reproducible. The analysis time is short (6·5 min) and there is a good separation between the $HbA_2$ values of β-thalassemia carriers from normals and α-thalassemia carriers, with no overlap between these groups. In addition, the system is also able to detect and quantitate most of the haemoglobin variants, particularly those (HbS, HbC, HbE and Hb Lepore) able to interact with β-thalassemia and could make haemoglobin electrophoresis unnecessary in all samples. The ease of operation and the limited technical work make this system especially suitable for laboratories with a high workload and allow the cost of screening to be reduced.*

### Introduction

Quantitative haemoglobin $HbA_2$ determination is a critical test for identifying carriers of β-thalassemia, because the increase of this minor haemoglobin fraction is the most relevant diagnostic characteristic of heterozygous β-thalassemia. Several laboratory techniques have been developed to measure accurately the $HbA_2$ levels [1-7], but they are all time-consuming manual methods and measure $HbA_2$ only—a complete haematological evaluation requires other tests: for example electrophoresis on different substrates, alkali denaturation for HbF, and elution or chromatography for quantitation of haemoglobin variants.

The introduction of a fully automated HPLC system for qualitative and quantitative haemoglobin analysis has produced a substantial improvement in the authors' laboratory [8]. The system performed separation and quantitative determination of haemoglobin types from whole blood. Although the method is accurate and reproducible, there were several problems to be overcome. These problems included difficult calibration of the instrument, the need for manual modification and installation of the program and the long analysis time (16 min/sample). Recently, a new version of this system was introduced and the results have improved considerably. This paper reports on the use of this system in a screening program for thalassemia.

### Materials and methods

The study involved 823 Sardinian adults who were examined as part of a screening program for thalassemia, in addition there were 13 subjects who were known to be carriers of haemoglobin variants. Red blood cell indices were determined with the Coulter Counter Max M. (Coulter Electronic) and haemoglobin analysis and quantitation were performed by HPLC VARIANT (Bio-Rad Laboratories, Milan, Italy). The VARIANT is a fully automated HPLC apparatus with a temperature controlled cation-exchange analytical cartridge (30 × 4·6 mm) and an increasing ionic strength elution buffer for a differential separation of haemoglobin components. A dual wavelength filter photometer (415 and 690 nm) reads the haemoglobins eluted from the cartridge. For the analysis, 5 μl of EDTA whole blood is automatically diluted with 1 ml of a haemolysing reagent. Haemolysed specimens are loaded into a 100-place sampler compartment maintained at $12 \pm 2$°C. Twenty microlitres of each sample are sequentially injected at 6·5 min intervals. Built-in software controls the analysis cycle (elution gradient, column regeneration) and performs peak integration. The calibration factors for $HbA_2$ and F are automatically calculated using a calibrator at the beginning of each run. The control program for the instrument is upgraded with an interface card.

Haemoglobin electrophoresis was performed on cellulose acetate in TrisEDTA borate buffer at pH 8·4, when a haemoglobin variant was detected citrate agar at pH 6·0 was used.

Globin chain synthesis was carried out on peripheral blood reticulocytes [9]. The α-globin genotype was defined by methods based on polymerase chain reaction (PCR), according to Dodé *et al.* [10] and Bowden *et al.* [11]. The haemoglobin variants were identified at DNA level by direct sequencing of β and δ globin genes after amplification by PCR [12, 13].

### Diagnostic criteria

Subjects with reduced MCV and MCH and increased (>3·5%) $HbA_2$ were classified as β-thalassemia carriers, while subjects with reduced MCV and MCH, normal serum iron and normal $HbA_2$ and F were classified as α-thalassemia carriers. The diagnosis of the α-thalassemia trait was confirmed in a large majority of cases by globin

* A. Mosca is at the Dipartimento Scienze e Tecnologie Biomediche, Università degli Studi di Milano, Via Olgettina, 60; 20132 Milan, Italy. Correspondence to Renzo Galanello, Istituto di Clinica e Biologia dell'Età Evolutiva, Ospedale Regionale Microcitemie USL 21, Università degli Studi di Cagliari, Via Jenner s/n, 09100 Cagliari, Italy.

0142-0453/95 $10.00 © 1995 Taylor & Francis Ltd.

*Table 1. Analytical imprecision for $HbA_2$ and HbF.*

| | $HbA_2$, % | | | HbF, % | | |
|---|---|---|---|---|---|---|
| Within run | $N$ | Mean ± SD | CV% | $N$ | Mean ± SD | CV% |
| Low | 15 | 1·96 ± 0·05 | 2·5 | — | — | — |
| Normal | 15 | 2·95 ± 0·04 | 1·7 | — | — | — |
| High | 15 | 4·91 ± 0·05 | 2·0 | 15 | 7·11 ± 0·08 | 1·2 |
| Between run | $N$ | | | | | |
| Low | 10 | 2·09 ± 0·03 | 1·4 | — | — | — |
| Normal | 10 | 3·10 ± 0·04 | 1·4 | — | — | — |
| High | 10 | 7·00 ± 0·11 | 1·6 | 10 | 6·95 ± 0·12 | 1·7 |

synthesis ($\alpha/\beta$ ratio $< 0{\cdot}9$ in 33 subjects) or $\alpha$ globin gene analysis (identification of deletion or non deletion defects, in 42 subjects).

### Results

In a previous paper [8], the authors reported the high reproducibility and accuracy of $HbA_2$ and F determination comparing the Diamat HPLC analyser with the DE-52 microchromatography for $HbA_2$ and with the alkali denaturation for HbF. The coefficient of variation within-run was 2·6% for $HbA_2$ and 5·1% for HbF; the correlation was for $HbA_2$: $r = 0{\cdot}9639$ and for HbF: $r = 0{\cdot}9990$.

The accuracy of $HbA_2$ (237 samples) and HbF (44 samples) measurements by the Variant analyser was established by taking the Diamat-HPLC analyser as reference method—see figure 1. Figure 1 uses the standard mode of reporting this type of data (the results of method No. 1 versus the results of method No. 2, top part of the figure), as well as the method proposed by Bland and Altman (bottom) [14]. The mean differences between the two analysers were −0·01% ($HbA_2$; 95% confidence intervals +0·31/−0·33%) and −0·22% (HbF; 95% confidence intervals +0·64/−1·08%). In conclusion, there was good agreement between the two methods, both for $HbA_2$ and HbF.

*Table 2. $HbA_2$ and F in normal subjects and in thalassemia carriers (mean ± SD).*

| | $N$ | $A_2$% | F% |
|---|---|---|---|
| Normal | 566 | 2·5 ± 0·2 | 0·6 ± 0·4 |
| $\beta$-trait | 163 | 5·5 ± 0·5 | 1·3 ± 1·4 |
| $\alpha$-trait | 94 | 2·4 ± 0·2 | 0·5 ± 0·4 |

The analytical imprecision was tested for $HbA_2$ by running several samples from subjects with low, normal and high $HbA_2$ levels, and separately from a sample with increased HbF. The results, reported in table 1, show that the $HbA_2$ and HbF determination is highly reproducible, with the coefficient of variation never greater than 3%.

Table 2 summarizes the values of $HbA_2$ and F found in a large group of normal subjects, $\beta$ and $\alpha$-thalassemia carriers. The cut-off limit for $HbA_2$ can be set at 3·5%, with all subjects with values higher than 3·5% being identified as $\beta$-thalassemia carriers.

In this study subjects with different haemoglobin variants of the $\alpha$ (Hb J Sardegna), $\beta$ (S, C, G San José, E, G Copenhagen, D, Shelby, Hope, Olbia) and $\delta$ ($A_2$ B, $A_2$ Sant'Antioco, Babinga, $A_2$ Fitzroy) chains were also examined. The nucleotide substitution of these variants have been defined by globin gene DNA sequencing. Figure 2 shows some chromatograms of these variants and figure 3 shows a diagrammatic representation of the relative positions of some common haemoglobin variants in the chromatogram. While HbS (figure 2 (*a*)) and C are eluted separately after $HbA_2$, Hb Lepore (figure 2(*b*)) and HbE are co-eluted with $HbA_2$. In these cases the percentage of the peak in the $HbA_2$ position will be greater than

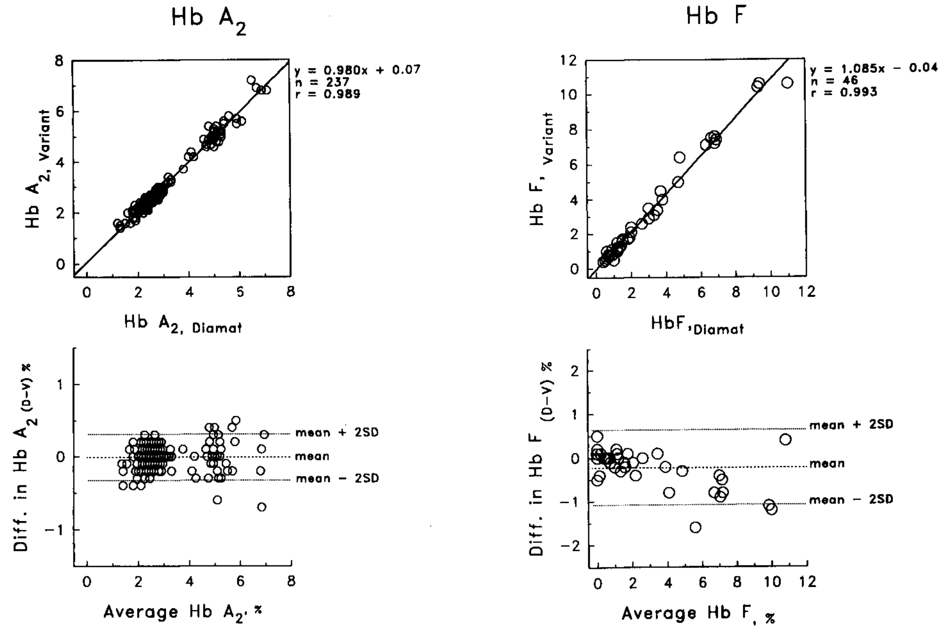

*Figure 1. Comparison between Diamat and Variant determinations of $HbA_2$ (left) and HbF (right), both expressed in percentages.*

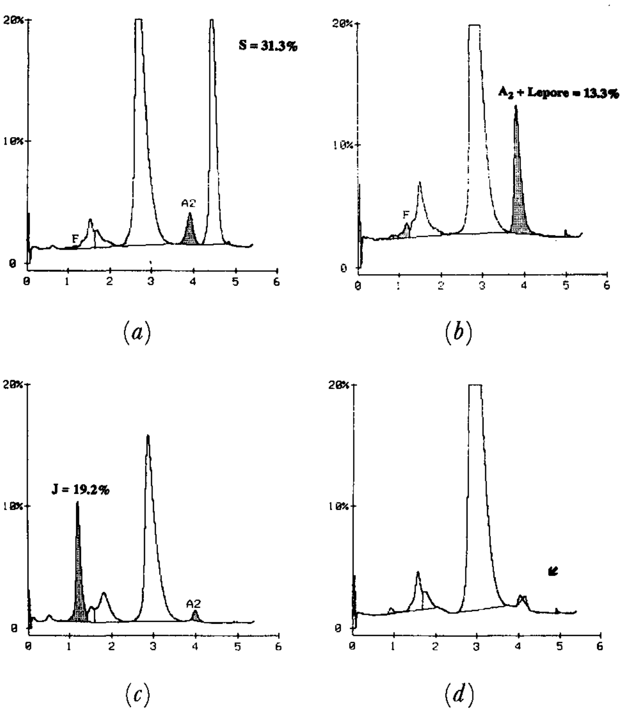

*Figure 2. Chromatograms from subjects with haemoglobin variants: (a) HbS; (b) Hb Lepore; (c) HbJ Sardegna and (d) $HbA_2$ S. Antioco.*

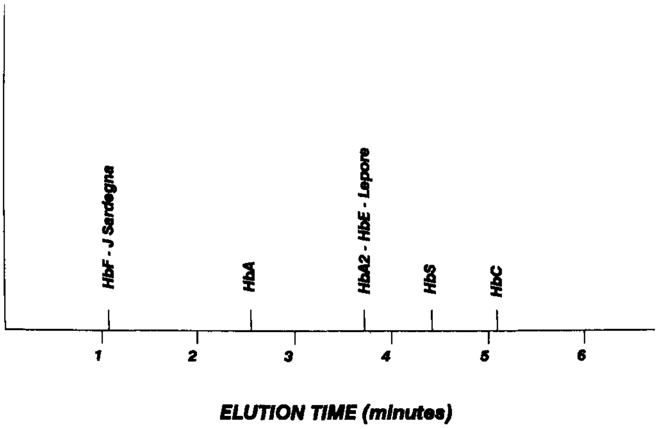

*Figure 3. Diagrammatic representation of the relative chromatographic position of some haemoglobin variants.*

10%. In HbS carriers there is a false increase of $HbA_2$ levels in the range 4·0–4·7%. Hb J Sardegna ($\alpha$ His → Asp) (figure 2[*c*]) shows an elution time similar to HbF. Electrophoresis on cellulose acetate will clear up the difference between these two haemoglobins, in fact HbJ Sardegna is an electrophoretically fast-moving variant. Figure 2(*d*) shows a double peak near the $HbA_2$ position due to the presence of an $HbA_2$ variant ($HbA_2$ S.Antioco $\delta$ 93 Lys → Gly) and of the normal $HbA_2$ [15]. With the Variant system, patients homozygous for $\beta^{\circ}39$ mutation do not show any peak correspondent to the $HbA_0$ elution time, as expected (not shown). However, with the Diamat a small peak in the $HbA_0$ position was found in $\beta^{\circ}$ homozygotes which is either an artefact or an unidentified component [8]. All the chromatograms are clear and easily understandable.

### Discussion

The accurate determination of $HbA_2$ and F, and the detection of the haemoglobin variants, usually require time-consuming methods. The Variant HPLC system provides a rapid, simple and reliable separation and determination of the relative percentage of different haemoglobin types, particularly haemoglobin $A_2$ and F, in whole blood. The method is accurate and reproducible. Other advantages are minimal sample preparation (5 µl of whole blood diluted automatically 1:200 in a single step), a short analysis time (6·5 min per sample), and the ability of the autosampler to analyse up to 100 samples sequentially and automatically. There is a good separation of the $HbA_2$ values among $\beta$-thalassemia carriers, normals and $\alpha$-thalassemia carriers, with practically no overlap between these three groups.

With regards to the detection limits, because of the lack of pure $HbA_0$, $HbA_2$ and HbF, it was not possible to perform any specific test so the limits claimed by the manufacturer, which were set at 0·7%, both for $HbA_2$ and HbF, were used.

The system is also able to detect and quantitate most of the haemoglobin variants and could make haemoglobin electrophoresis, commonly used in haemoglobinopathies screening, no longer necessary in all samples. However, for the identification of any particular haemoglobin variant, other methods (like sickling test for HbS, electrophoresis on different substrates, globin chain analysis, instability tests, protein analysis or DNA sequence analysis) are required.

There are some limitations in the procedure. Since Hb Lepore and HbE are co-eluted with $HbA_2$, their presence in the sample will give a percentage of $HbA_2$ which is greater than 10%. This amount of $HbA_2$ is almost never present in $\beta$-thalassemia carriers. Therefore samples found to have a level of $HbA_2$ greater than 10% should be further tested for the possible presence of a haemoglobin variant interference. The false increase of $HbA_2$ levels in HbS carriers is due to the co-elution of minor components with $HbA_2$ (possible post-translational modifications of HbS). This may also occur with haemoglobin variants eluting after $HbA_2$. HbH ($\beta_4$) and Bart's ($\gamma_4$) can be detected in the chromatogram but not quantitated, because they are eluted prior to the start of integration.

The ease of operation and the limited technical work make this system especially suitable for laboratories with a high workload; it will also reduce the screening costs for $\beta$-thalassemia and haemoglobinopathies.

### Acknowledgements

The authors would like to thank Professor A. Cao (Università degli Studi, Cagliari, Italy) for helpful discussions and suggestions; and M. Loi, G. Corda, V. Demurtas, and I. Curreli for skillful technical assistance, M. Furbetta and A. Piga for some of the Hb variants samples, and S. Longoni and F. Fodde for editorial assistance. This work was supported by the CNR-Target project Ingegneria Genetica, subproject

Diagnosi molecolare di talassemia intermedia N.92.00431.-pf 99, Diagnostica delle Talassemie: organizzazione e standardizzazione della diagnosi prenatale, N.91.04193.-ST75, MPI 60%, and Legge Regionale 30.04.1990 n.11 Regione Sardegna.

### References

1. Williard, R. F., Lovell, W. J., Dreiling, B. J. and Steinberg, M. H., *Clinical Chemistry*, **9** (1973), 1082.
2. International Committee for Standardization in Haematology (ICSH), *British Journal of Haematology*, **38** (1978), 573.
3. Schmidt, R. M. and Brosious, E. M., *American Journal of Clinical Pathology*, **71** (1979), 534.
4. Galanello, R., Melis, M. A., Muroni, P. and Cao, A., *Acta Haematologica*, **57** (1977), 32.
5. Schleider, C. T., Mayson, S. M. and Huisman, T. H., *Hemoglobin*, **1** (1977) 503.
6. McCormack, M. K., *Clinica Chimica Acta*, **105** (1980), 387.
7. Garver, F. A., Singh, H., Moscoso, H., Kestler, D. P. and McGuire, B. S., *Journal of Clinical Chemistry*, **30** (1984), 1205.
8. Mosca, A., Carpinelli, A., Majavacca, R., Cantu'-Rainoldi, A., Garatti, M., Paleari, R., Ferrari, M., Agape, V., Maccioni, L., Pisano, S. and Galanello, R., *Journal of Automatic Chemistry*, **11** (1989), 273.
9. Kan, Y. W., Schwartz, E. and Nathan, D. G. J., *Clinical Investigation*, **47** (1986), 2515.
10. Dode', C., Krishnamoorthy, R., Lamb, J. and Rochette, J., *British Journal of Haematology*, **82** (1993), 105.
11. Bowden, D. K., Vickers, M. A. and Higgs, D. R., *British Journal of Haematology*, **81** (1992), 104.
12. Mullis, K. B., Faloona, F. A., Scharf, F. A., Saiki, R. K., Horn, G. T. and Erlich, H. A., *Cold Spring Harbor Symposia on Quantitative Biology*, **51** (1986), 263.
13. Sanger, F., Nicklen, S. and Coulson, A. R., *Proceedings of the National Academy of Sciences of the United States of America*, **74** (1977), 5463.
14. Bland, J. M. and Altman, D. G., Statistical methods for assessing agreement between two methods of clinical measurement. *Lancet*, **i** (1986), 307.
15. Galanello, R., Gasperini, D., Perseu, L., Barella, S., Ideo, A. and Cao, A. $HbA_2$—S.Antioco ($\alpha_2\ \delta_2$ 93 (F9) Cys → Gly): a new $\delta$ chain VARIANT identified by sequencing of amplified DNA. *Hemoglobin*, **18** (1994), 437.

